# Research on epileptic EEG recognition based on improved residual networks of 1-D CNN and indRNN

**DOI:** 10.1186/s12911-021-01438-5

**Published:** 2021-07-30

**Authors:** Mengnan Ma, Yinlin Cheng, Xiaoyan Wei, Ziyi Chen, Yi Zhou

**Affiliations:** 1 School of Biomedical Engineering, Sun Yat-sen University, No.132 Waihuan East Road, Guangzhou, 510006 China; 2 Department of Medical Informatics, Zhongshan School of Medicine, Sun Yat-sen University, No.74 Zhongshan 2nd Road, Guangzhou, 510080 China; 3 Minister of Science, Education and Data Management Department, Guangzhou Women and Children’s Medical Center, National Children’s Medical Center for South Central Region, Guangzhou Medical University, No.9 Jinsui Road, Guangzhou, 510623 China; 4 Department of Neurology, First Affiliated Hospital, Sun Yat-sen University, No.58 Zhongshan 2nd Road, Guangzhou, 510080 China; 5 Key Laboratory of Tropical Disease Control (Sun Yat-sen University), Ministry of Education, No.74 Zhongshan 2nd Road, Guangzhou, 510080 China

**Keywords:** Epilepsy, Residual network, CNN, indRNN, RCNN

## Abstract

**Background:**

Epilepsy is one of the diseases of the nervous system, which has a large population in the world. Traditional diagnosis methods mostly depended on the professional neurologists’ reading of the electroencephalogram (EEG), which was time-consuming, inefficient, and subjective. In recent years, automatic epilepsy diagnosis of EEG by deep learning had attracted more and more attention. But the potential of deep neural networks in seizure detection had not been fully developed.

**Methods:**

In this article, we used a one-dimensional convolutional neural network (1-D CNN) to replace the residual network architecture’s traditional convolutional neural network (CNN). Moreover, we combined the Independent recurrent neural network (indRNN) and CNN to form a new residual network architecture-independent convolutional recurrent neural network (RCNN). Our model can achieve an automatic diagnosis of epilepsy EEG. Firstly, the important features of EEG were learned by using the residual network architecture of 1-D CNN. Then the relationship between the sequences were learned by using the recurrent neural network. Finally, the model outputted the classification results.

**Results:**

On the small sample data sets of Bonn University, our method was superior to the baseline methods and achieved 100% classification accuracy, 100% classification specificity. For the noisy real-world data, our method also exhibited powerful performance.

**Conclusion:**

The model we proposed can quickly and accurately identify the different periods of EEG in an ideal condition and the real-world condition. The model can provide automatic detection capabilities for clinical epilepsy EEG detection. We hoped to provide a positive significance for the prediction of epileptic seizures EEG.

## Background

Epilepsy is a chronic brain dysfunction syndrome. Nearly 65 million people in the world are suffering from epilepsy, which accounts for about 1% of the world’s population [1]. The causes of epilepsy were various, and the course of the disease would repeat for a long time. So that, epilepsy not only seriously endangered the health of patients but also brought great mental pressures [2, 3]. The development of EEG provided a non-invasive, low-cost, and effective technology that can be used in clinical trials to detect cerebral cortex brain activity and related diseases [4–6]. A large number of studies have shown that epilepsy EEG was significantly different from normal EEG. The brain activities of patients with epilepsy usually included the interictal and the ictal period [7]. When brain activities change from one state to another, the EEG signal will change obviously. Therefore, EEG is an important basis for the clinical diagnosis of epilepsy.

The professional neurologists’reading of EEG is the main method to determine epilepsy [8]. However, it is complex and time-consuming to observe and detect long-range EEG signals by people. The heavy workload can easily cause fatigue of medical personnel and lead to inaccurate manual detection. Moreover, we often make a judgment only through professional experience. So, an automatic detection and classification model of EEG become more and more urgent and important.

In recent years, some significant progress has been made in the diagnosis of epileptic diseases and the detection of seizures based on Electroencephalogram (EEG). Nonlinear dynamics, machine learning, deep learning and other technologies have also been applied to the research of epileptic diseases, and good results have been achieved. The recurrent neural network(RNN) [9] and the CNN [10] are the research hotpots in recent years. The former focuses on the context of time series, and the latter focuses more on EEG feature extraction. Unlike traditional small-dose calculation research, deep neural networks are focused more on big data research. However, there are still some problems that cannot be solved at this stage.

The main contributions of our work are as follows: a new neural network architecture, which was the recurrent convolution neural network(RCNN), was proposed by combining the advantages of the recurrent neural networks and the convolutional neural networks. This new architecture used the one-dimensional convolutional neural network to extract the features of the original EEG, then used the independent recurrent neural network to learn the relationship between time series, and finally realized the automatic diagnosis of epileptic EEG by classification function. We used the Bonn University data sets and our private data sets for training and testing. In order to avoid the individual specificity in the epilepsy diagnosis algorithm, we ensured that the data sets used in the experiment were from different individuals, and compared with the relevant frontier research. Our method had generally achieved good results. In the binary classification task, even 100% of results are obtained in accuracy and specificity. These results indicated that the new architecture we proposed can effectively realize the automatic detection of epilepsy EEG.

## Related work

### Models of convolutional neural network

**Fig. 1:**
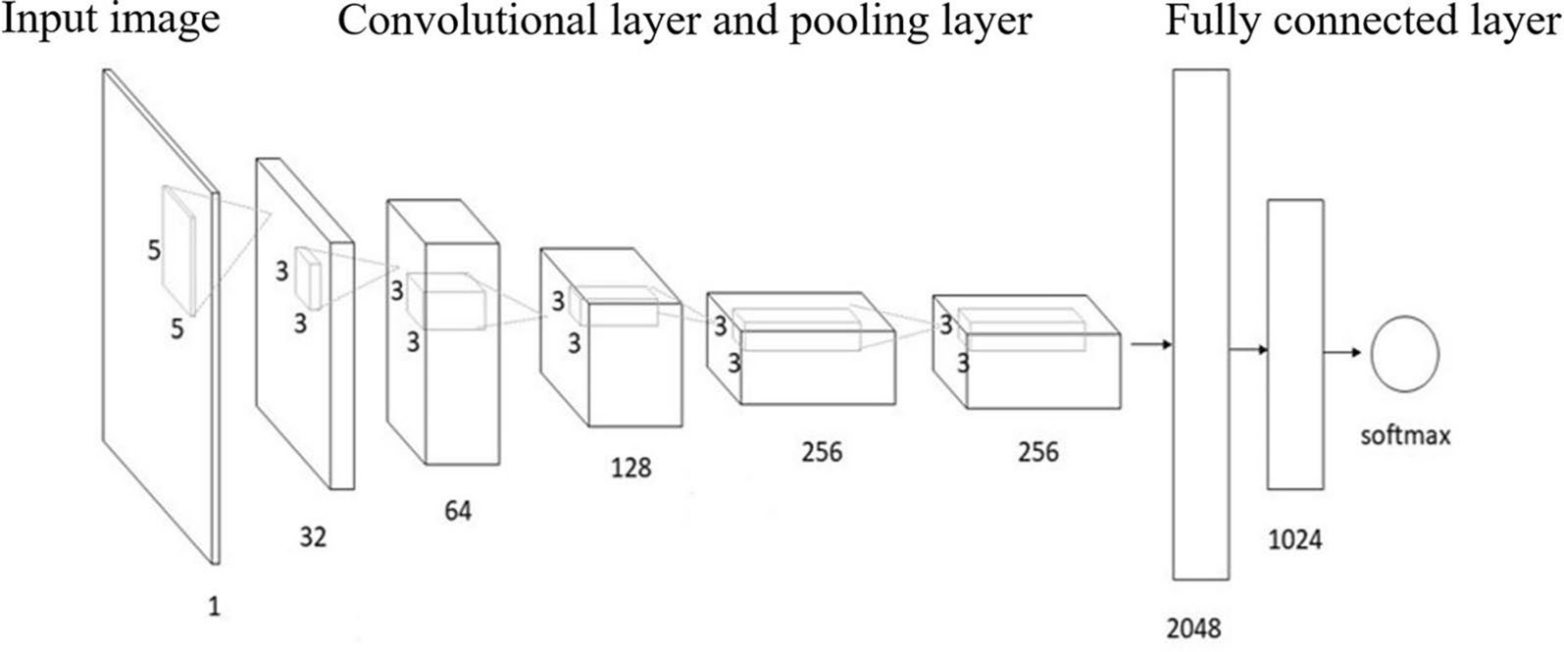
Schematic diagram of convolutional neural network structure

Convolutional neural network (CNN) is a type of feed-forward neural network that includes convolution operations and has a deep structure. The characteristics of it are local perception and parameter sharing, which is one of the hottest research points in the field of image processing [11]. A complete CNN contains a convolution layer, a down-sampling layer, and a fully connected layer [12], as shown in Fig. 1. The convolution layer can enhance certain features of the original EEG signal through convolution operations and reduce the impact of noise; The downsampled layer, which is often referred to as the pooling operation, can reduce the amount of data processing while retaining useful Information; the fully connected layer gets the image features extracted by the neural network. And the full connection layer outputs the final result through the function “softmax” in Fig. 1.

**Fig. 2:**
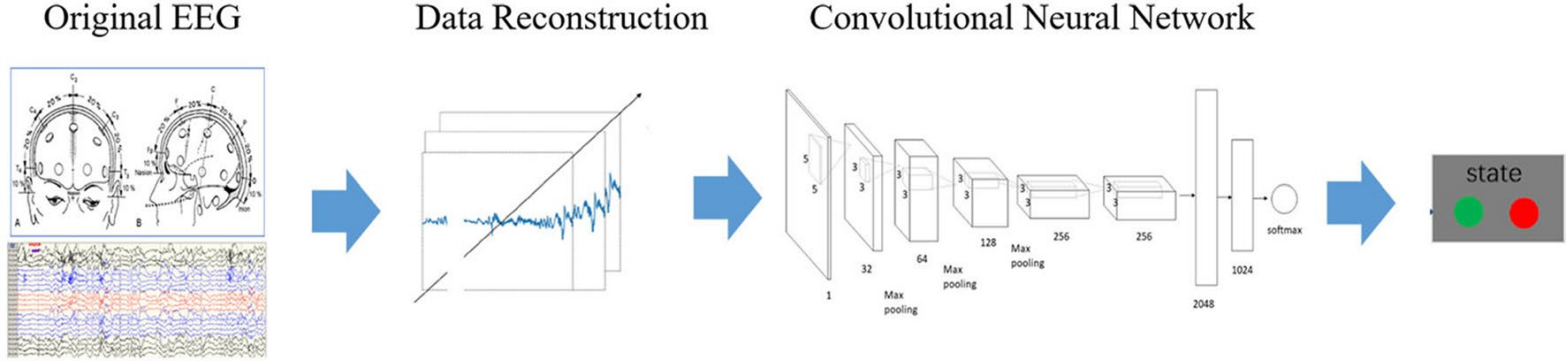
10/20 system electrode placement

Figure 2 shows a common frame diagram of epilepsy prediction system based on convolutional neural network. The key of the system lies in the reconstruction of the original EEG data, that is, the reconstruction of the input image. In the existing methods, one-dimensional, two-dimensional and three-dimensional EEG are reconstructed according to the dimension of convolution operation [13–17]. The latest seizure prediction studies which use CNN in recent years, use convolution features of different dimensions to build models. Convolutional neural network often uses reconstructed image processing in the prediction of seizures. With the changes of EEG data dimension, important information may be lost. The existence of the pooling layer will also lead to the loss of many very valuable information. And it will also ignore the relationship between the whole and the part. Therefore, the research based on convolution model need to be improved.

### Models of recurrent neural network

**Fig. 3:**
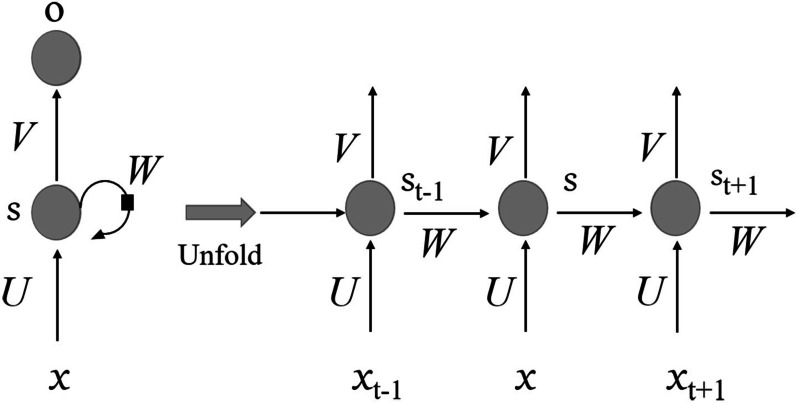
Schematic diagram of recurrent neural network structure

Recurrent neural networks (RNN) have achieved great success and are widely used in the field of time series analysis [18], for example, the natural language processing. The recurrent neural network includes an input layer, a hidden layer and an output layer. Figure 3 shows a complete recurrent neural network structure. Among them, *x* is the input data; *s* is the memory of the sample at time *t*, that is, the hidden layer; *o* is the output sample, and *U* and *V* are the weight of the input and output samples. Different from the traditional feedforward feedback neural network, RNN introduces a directional loop, as shown in the right part of Fig. 3. The output of time *t* is related to the previous time as well as the current time. RNN can deal with the problem of correlation between inputs, especially the EEG time series analysis.

Different from the EEG analysis of convolutional neural network, the original EEG data does not need to be reconstructed, and RNN can directly process EEG sequence, which ensures the maximum information retention of EEG. CNN has obvious advantages, but there are still shortcomings, and the situation of RNN is similar. Because the parameters of each recurrent layer network are shared, as the depth of the layer increases, the RNN will have the problem of gradient explosion or gradient disappearance. In order to solve these problems, some variant RNNs, such as GRU [19], LSTM [20], bi-LSTM [21] and many more are used in the study of seizure prediction [22–24].

### Models of recurrent convolution neural network

**Fig. 4:**
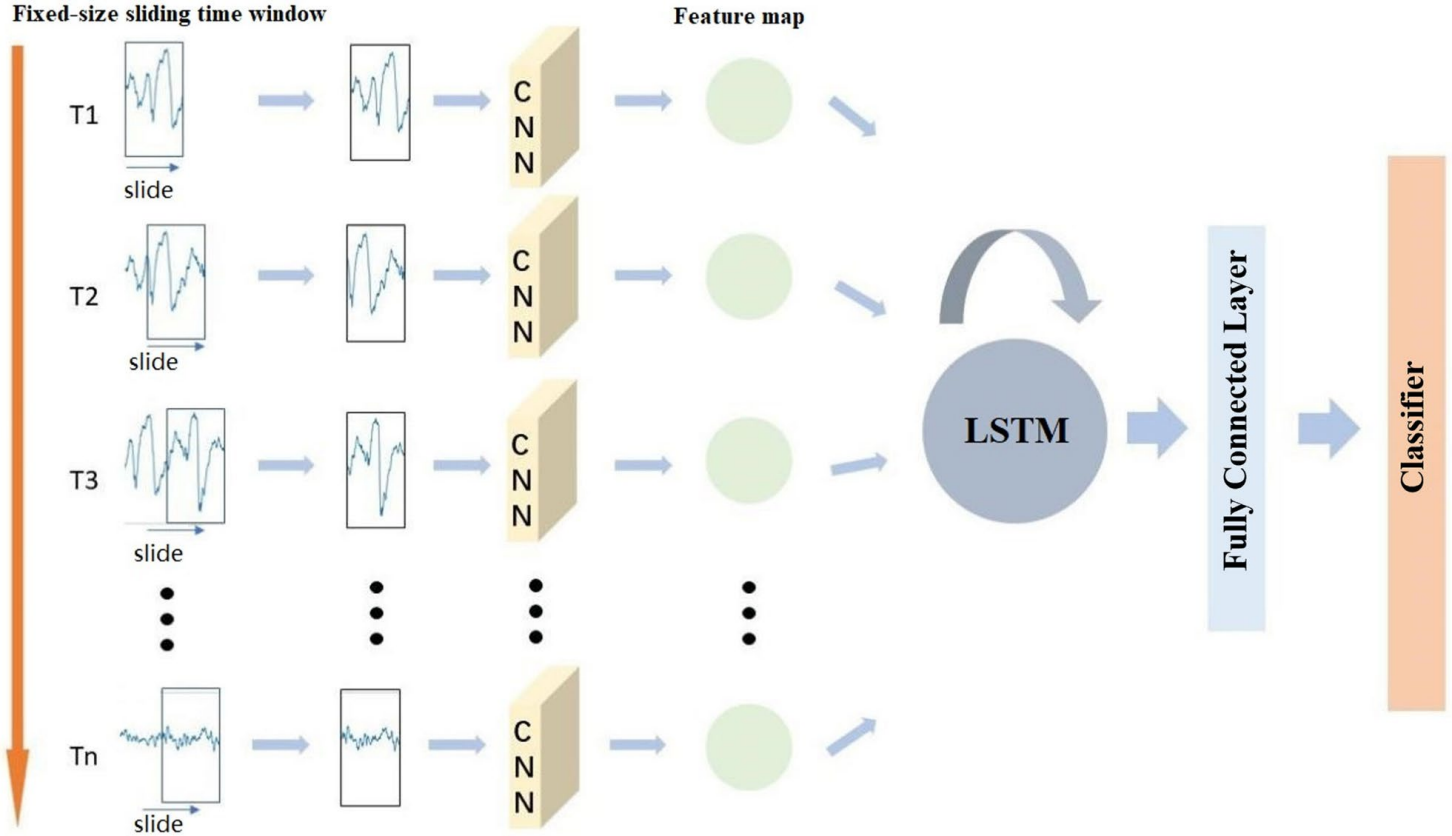
Framework diagram of epileptic seizure prediction using Recurrent Convolutional Network

We listed the researches of CNN and RNN in epileptic seizure. CNN has better spatial information capturing ability, while RNN is better at analyzing the relationship between time series. We know that EEG collected from the clinic is not only the cumulative of time, but also the interaction between different leads. And the spatial relationships between brain network nodes are equally important. Considering the different advantages of CNN and RNN, a new architecture idea is applied to EEG analysis. Figure 4 shows a CNN and RNN recurrent convolutional neural network seizure detection system. The basic idea is that under the movement of a fixed time sliding window, the CNN network learns the spatial features between the sequences and extracts them. And the network inputs them into the recurrent neural network according to the time sequence. Finally, the classification results are given through the time relationship between RNN learning sequences.

The research based on this new architecture has not been carried out sufficiently. Ahmedt David et al. [25] proposed a simple end-to-end architecture based on convolution and recurrent neural networks, which extracted spatiotemporal representations from 119 epileptic seizure databases of 28 patients. Subsequently, the cosine similarity distance between the test representation and the library of five abnormal seizures separated from the test data set was used to identify seizure tests with abnormal patterns that did not conform to known behaviors. The motion features extracted based on clinical image data were used for seizure detection to provide more possibilities for the diagnosis of epilepsy diseases. Although this framework did not use EEG data, it was possible for multimodal research to predict seizures based on EEG and Surveillance Video.

Meysam et al. [26] proposed a seizure detection model of convolution and long-short-term memory recurrent neural network based on clinical detection of EEG. This model can input the convolution features of the multi-channel EEG data of the reconstructed two-dimensional pictures into the relationships between the learning sequences in the LSTM, and finally output the classification results. XY Wei et al. [27] proposed a similar study, but the difference was the image reconstruction. In this paper, the team converted the EEG time series into two-dimensional EEG, and then fused multi-channel into three-dimensional structure. A feasible method, long-term recursive convolutional network (LRCN), was proposed to implement an end-to-end automatic prediction model for seizures.

Convolutional network blocks are used to automatically extract depth features from data. The LRCN combined the LSTM neural network block to distinguish different image sequences and identified the front segment from the streaming data. The model was tested with independent data and provided a higher sensitivity of seizure prediction and a low false prediction rate of 0.04 FP/h than the method manually designed in previous studies and a single deep neural network. This reconstruction of the external structure was an important point of our researches, but some teams considered the combination of two networks within the neural network. The team of Li Feifei [28] proposed an E3D-LSTM network with strong memory. 3D convolution was used instead of 2D convolution as the basic calculation operation of LSTM network, and a self-attention mechanism was added to enable the network to take into account both long-term and short-term information dependence and local spatiotemporal feature extraction. This provided new ideas for video prediction, motion classification and other related issues, and was a very enlightening work.

## Materials and methods

### Data

#### Public dataset

The public data set used in this experiment is from the EEG data set of the University of Berne [29]. This data set has been preprocessed into EEG under ideal conditions and is widely used. The database contains five groups, each group of 100 time series, each time series length is 23.6 s. Groups A and B are from the resting state of healthy volunteers, Groups C, D, and E all come from patients’ EEG. The difference is that the time of epileptic seizure is recorded in Group E, while there is no seizure in the other two groups during the whole recording period.

#### Private dataset

The private data set for this experiment came from the Department of Neurology at the First Affiliated Hospital of Xinjiang Medical University and the First Affiliated Hospital of Sun Yat-Sen University from 2013 to 2016. There were 15 patients with epilepsy (5 males and 10 females, aged 6–51 years). Scalp electrodes are placed according to the international 10–20 system. The sampling frequency is 500 Hz. The total duration of available EEG recordings is approximately 540 h. The onset and offset time intervals are manually annotated by clinical experts after visual inspection, for a total of 168 episodes. According to the requirements of experimental design and the definition of stages, EEG is classified into interictal period, preictal period, and ictal period. The information about the EEG data set is shown in Table 1.

**Table 1:** Experimental data specific information

ID	Sex	Age	Type	Time	Number of seizures	IT
1	F	36	SPS	48	10	654 s
2	F	22	SPS, CPS	48	12	274 s
3	F	36	CPS	48	14	1386 s
4	F	40	SPS	24	6	302 s
5	M	6	SPS	48	21	453 s
6	F	16	SPS, CPS	24	7	329 s
7	F	16	SPS, CPS	24	8	254 s
8	F	28	CPS	24	5	400 s
9	F	31	SPS	24	9	423 s
10	M	51	SPS	72	30	1064 s
11	M	20	SPS, CPS	48	19	4072 s
12	M	46	SPS	24	6	208 s
13	F	15	CPS	48	8	137 s
14	F	28	SPS	24	5	824 s
15	M	39	SPS, CPS	24	4	895 s

PS: F, female; M, male; SPS, simple partial; CPS, complex partial, IT, ictal time

#### Definitions of different periods

**Fig. 5:**
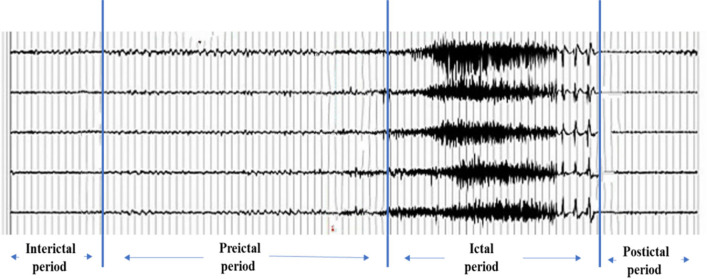
Distribution of core nodes in epilepsy leads

The seizure process is divided into four states, including interictal, preictal, ictal, postictal [30–32], as shown in Fig. 5. Clinical experts have marked the starting and ending points of seizures in the data. In order to predict future seizures, the key of the epileptic seizure prediction system is to separate preictal from the interictal period.

Preictal is defined as the data segment before the ictal (or seizure) cycle, which can be clearly identified from the EEG signal. However, different studies have different definitions of the length of preictal, ranging from 10 to 60 min [33]. In addition, each patient data contains at least two seizures in this study, but the interval between the two seizures can be long or short. Through the work of different studies, the best pre-onset period is now defined as 30 min before the onset. For seizures less than 30 min from the previous seizure, we think they are only one seizure.

Interictal is defined as the part of the signal that is neither ictal nor preictal. In our experiment, the data of at least 1 h after seizure and at least 40 min after seizure were defined as the interictal period through literature review. In this paper, the data of 30 min in the 35 min before each attack is used as the pre-onset data to maintain the balance of the positive and negative samples in the data sample set.

### Methods

#### System framework of RCNN

**Fig. 6:**
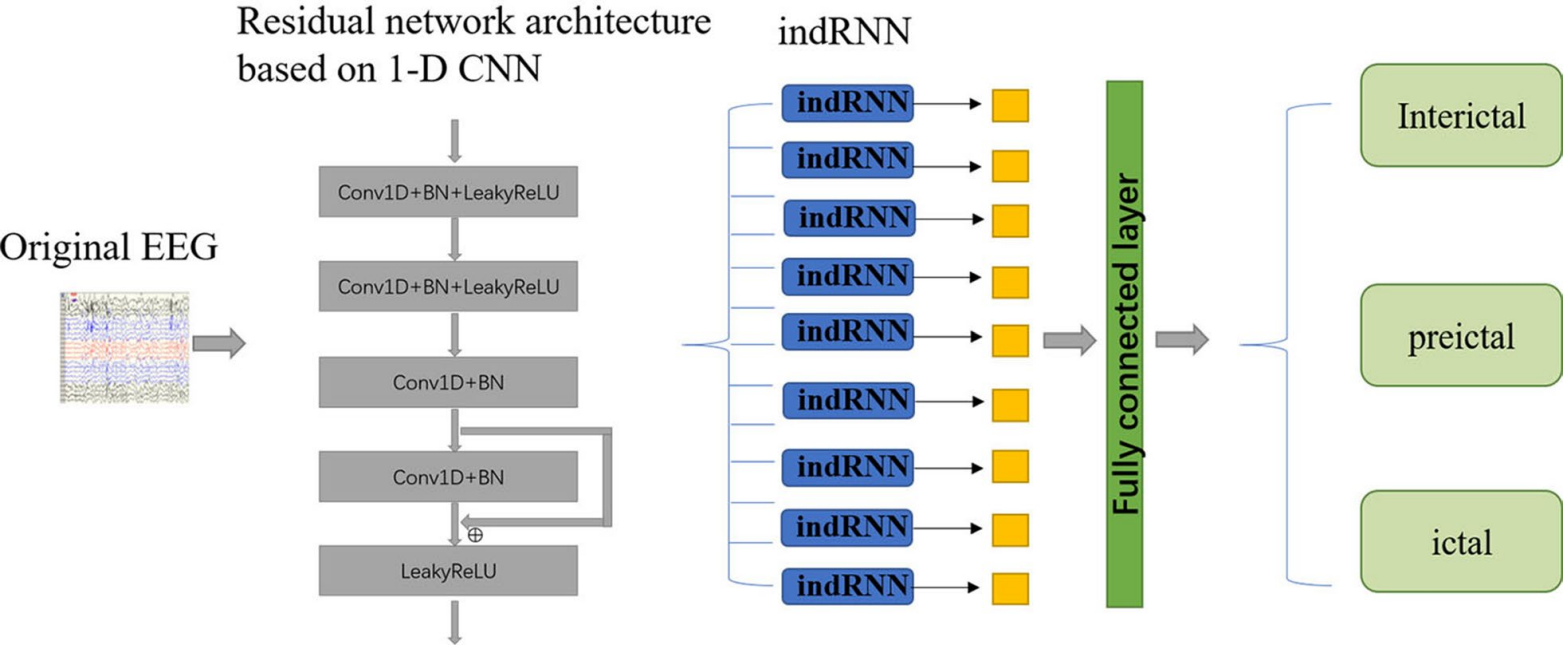
Different periods

In this study, the residual network architecture was combined with the recurrent neural network, and 1-D CNN was used to replace the two-dimensional convolution (2-D CNN) operation in the residual structure. The purpose of this was to establish a spatiotemporal deep learning model for epileptic seizure detection. 1-DCNN was used to automatically extract signal features from the original EEG, and the indRNN neural network was used to distinguish different categories based on the extracted features. The construction process of the epilepsy EEG automatic detection model in this paper is shown in Fig. 6. The model ran on a high-performance computer with Python 3.

In this study, the indRNN network was combined with the previously proposed convolutional neural network to create a spatiotemporal deep learning model for seizure detection. The traditional convolutional neural network processed the input data by convolution and pooling operations, and then inputted into the fully connected layer to output the results.

The input layer of our model was connected with a complete residual convolution neural network. The unreconstructed EEG signal was inputted into the convolutional neural network to output a transformed fixed-length feature vector. Multiple consecutive outputs formed a feature sequence, which is then input to indRNN. indRNN mapped the input to the hidden layer, updated the hidden layer, and finally outputted the predicted distribution result at time step t, and used softmax to determine the category. In this process, indRNN learned the changes of abstract features of EEG, and the output results determined the current EEG segment through the classification function, and then made a judgment.

#### Independent residual network architecture

Independent residual network architecture shown in the Table 2.

**Table 2:** Independent residual network architecture

Layer	Hidden layer	Related parameters (filters, kernels, stride)		
BLOCK1	Conv1D+BN+LeakyReLU	64	8	1
	Conv1D+BN+LeakyReLU	64	5	2
	Conv1D+BN	64	3	1
	Conv1D+BN	64	1	1
	Add	–	–	–
	LeakyReLU	–	–	–
BLOCK2	Conv1D+BN+LeakyReLU	128	8	1
	Conv1D+BN+LeakyReLU	128	5	2
	Conv1D+BN	128	3	1
	Conv1D+BN	128	1	1
	Add	–	–	–
	LeakyReLU	–	–	–
BLOCK3	Conv1D+BN+LeakyReLU	64	8	1
	Conv1D+BN+LeakyReLU	64	5	2
	Conv1D+BN	64	3	1
	Add	–	–	–
	LeakyReLU	–	–	–
	GlobalAveragePooling1D	–	2	–
	indRNN+BN	128		
	indRNN+BN	128		
	Fully connected	256		
	Softmax	n_class		

**Fig. 7:**
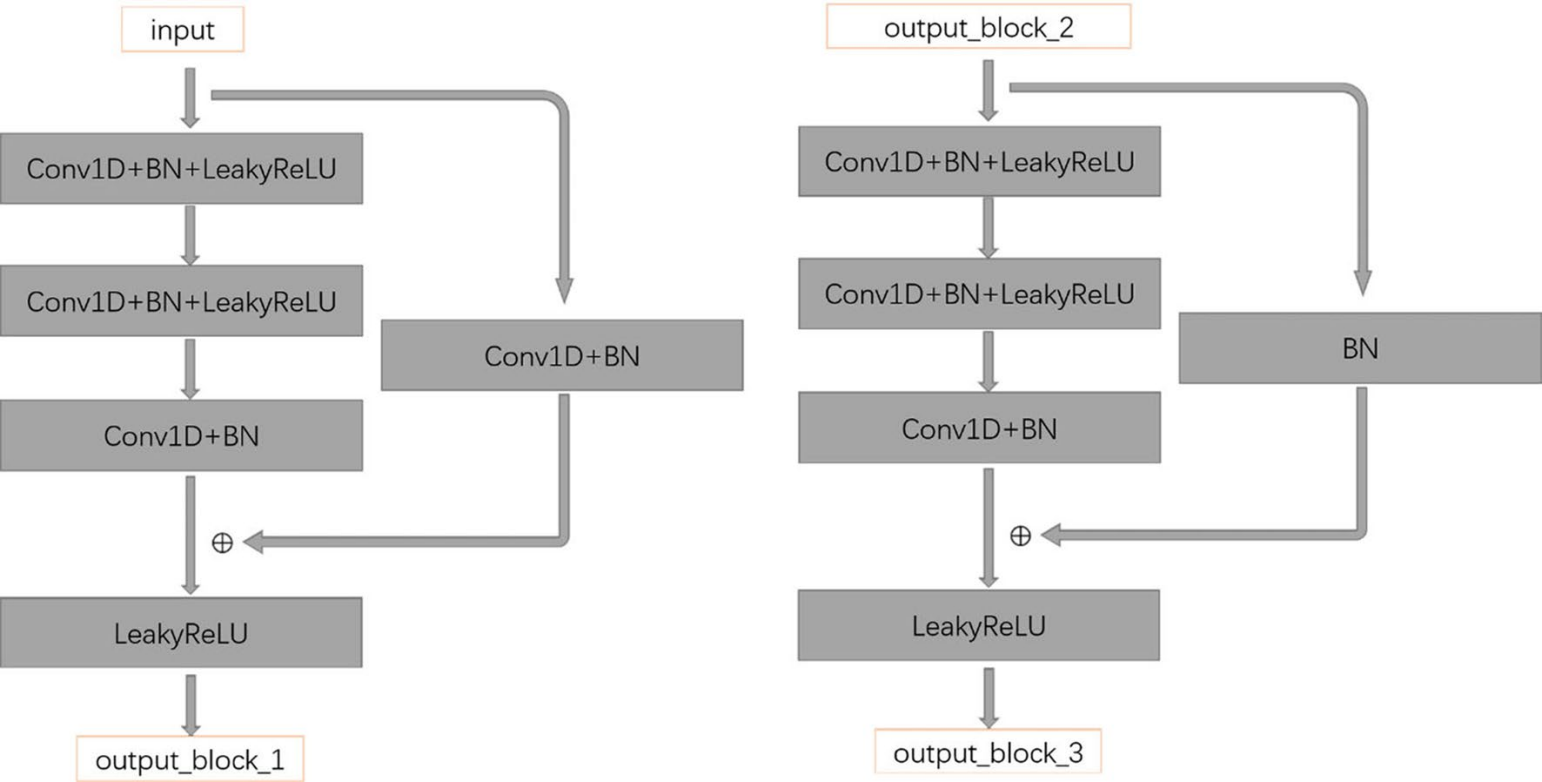
Residual network diagram

*Residual network* CNN can extract the features of low/mid/high-level. The more layers of the network, the richer the features of different levels can be extracted [34]. Moreover, the deeper the network is, the more abstract the features are and the more semantic information they have. If the layers behind the deep network are identity maps, then the model degenerates into a shallow network. After years of development, residual network has great advantages for deep-seated network learning. The residual architecture [35] used in this experiment is a convolutional network of three residual blocks, as shown in the Fig. 7 is the structure diagram of the first residual block, the stitching of the second residual block is the same as the first, the third as shown on the right, it is slightly different.

*Independent recurrent neural network* The recurrent neural network has been widely used in sequence learning problems such as action recognition, scene annotation, language processing, and has achieved remarkable results. Compared with feed-forward networks such as CNN, RNN has a cyclic connection, in which the last hidden state is the input to the next state. The training of RNN is faced with the problems of gradient vanishing and gradient explosion due to the multiplication of cyclic weight matrices. Therefore, it is actually difficult to construct and train deep LSTM or GRU based on RNN. Each neuron in IndRNN independently processes a type of spatiotemporal model [22]. Traditionally, RNN is regarded as a multi-layer perceptron with shared parameters in time. Different from the traditional RNN, the IndRNN neural network was used in this paper provides a new perspective for the recurrent neural network, that is, the spatial patterns are aggregated independently over time. The correlation between different neurons can be used by stacking two or more layers. In this case, each neuron in the next layer processes the output of all neurons in the previous layer which can solve the problems of gradient disappearance and gradient explosion.

*Network configuration* Our identification network was trained to global optimization by using “SGD” as the optimization function and “categorical_crossentropy” as the loss function. Stochastic Gradient Descent (SGD) was used to train various machine learning and deep learning models because of its fast learning speed and online updating. 2-D CNN in the residual network was used to replace by 1-D CNN, which can directly process the original EEG data without preprocessing the EEG dimension.

The parameters of the convolution unit were selected as: the length of convolution kernel was (8, 5, 3) and the number of convolution kernels was (64, 128, 128), the activation function was “LeakyReLU”. The convolution part was mainly to capture the short-term time correlation of EEG data; the part of circulating unit network was mainly used to distinguish EEG categories. With the increase of network depth, the gradient disappearance and gradient explosion problems of the recurrent network became more and more serious. In this experiment, the independent recurrent neural network was selected, and the number of neurons was set to 128. Finally, we used “Dense” to output the classification results, and selected the function “softmax” as the activation function. The batch size was set to 64, and the number of iterations was set to 512. We compared the public data set with the private data set, and various noise levels in the experiment are fully considered.

## Results and discussion

In this section, we mainly tested the epilepsy detection performance on public data sets with ideal conditions and real data sets with clinical trials. The sensitivity (Sens), specificity (Spec) and classification accuracy (Acc) of evaluation indicators were evaluated. The details will be described in the following paragraph.

### Experimental design

The difference between public data sets under ideal conditions and clinical experimental data sets was artifacts and noise, which will affect the detection results. We first tested on public data sets, and then tested on real data sets for comparison.

#### Model training and testing

**Fig. 8:**
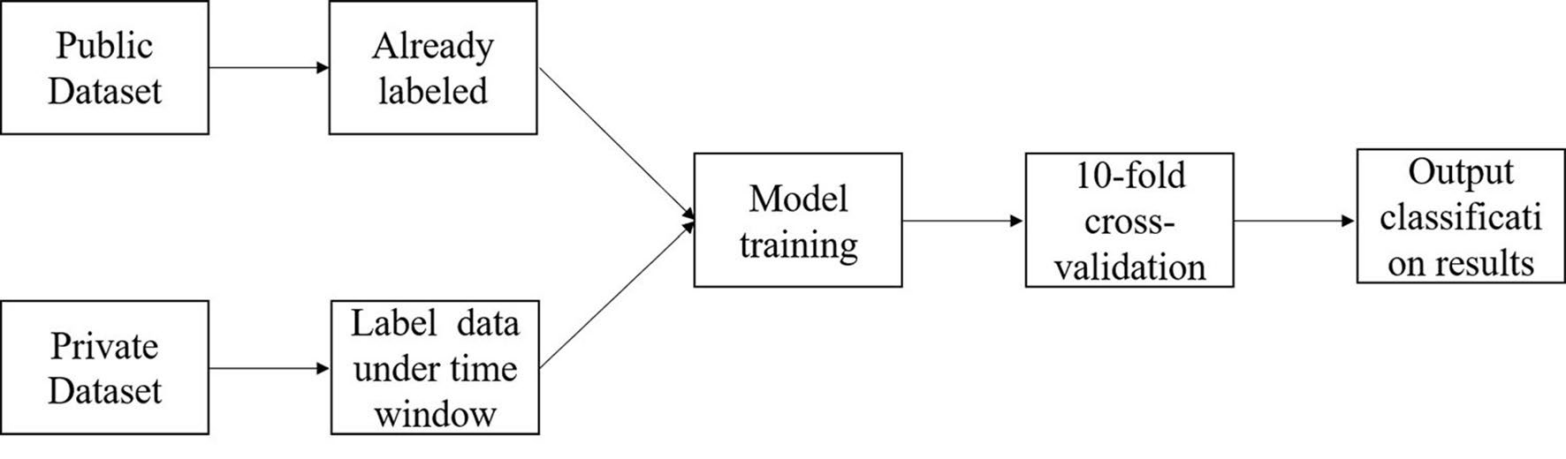
Training and testing of the experimental model

Epilepsy EEG classification research is the correct identification of EEG signal fragments, and the recognition of the epilepsy different periods which have already been defined. Figure 8 showed the training and testing of our experimental model. In this experiment, the open dataset contained three different kinds of EEG, which are the healthy people group, the epilepsy patients without seizure group and the epilepsy patients with seizure group. Therefore, according to the contrast principle, we designed two classification and three classification tasks to detect the performance of the algorithm. The experimental design of the two-class task was the EEG recognition of the healthy people group and the epilepsy patient group; the experimental design of the three-class task was the healthy people group and the non-seizure group and the seizure group.

In addition to the public dataset, our experiments took into account noise and interference in the real world, and selected specific types of epilepsy patients with interictal, preictal and ictal EEG for testing. Real data has the characteristics of streaming data. So in our experiments, we formalized it as windowed streaming data. Each 10s EEG segment was identified and the EEG category was output. Because this experiment does not involve the task of prediction, our EEG classification recognition only takes the time window, and discards the event classification task.

Ten folds cross-validation was used in this study. First, EEG signals were randomly divided into 10 equal parts. Nine out of ten EEG signals were used to train the neural network in this paper, and the remaining one-tenth were used to test the performance of the system. By moving the test and training data sets, the strategy was repeated ten times. The accuracy, sensitivity and specificity values reported in this paper are the average values obtained from ten evaluations.

#### Evaluation indicators

There are three most important and commonly used parameters to evaluate the performance of epileptic detection methods: sensitivity (Sen), specificity (Spe) and classification accuracy (Acc). Sen indicates the sensitivity of the recognition system, that is to say, it measures the ability of the classifier to capture EEG data. Spec indicates that the recognition system can correctly recognize the different periods EEG data, that is to say, it measures the classifier’s recognition ability for the different periods seizure. Acc indicates the recognition capability of the recognition system. In this article, sensitivity and specificity are equally important for the evaluation of our model. Sensitivity can reflect the missed diagnosis rate of our model, and specificity can reflect the misdiagnosis rate of our model. In our experiments, we did not pursue high specificity at the cost of sensitivity. On the contrary, we hope that we can find a balance between sensitivity and specificity. The specific formula is as follows:

1 $$\begin{aligned} Sen= & {} \frac{TP}{TP+FN} \end{aligned}$$

2 $$\begin{aligned} Spe= & {} \frac{TN}{TN+FP} \end{aligned}$$

3 $$\begin{aligned} Acc= & {} \frac{TP+TN}{TP+FN+TN+FP} \end{aligned}$$

Among them, *TP* is the number of correctly divided into positive examples, *FN* is the number of EEG that is wrongly divided into positive examples, *TN* is the number correctly divided into negative examples, and *FP* is the number of EEG that is wrongly divided into negative examples.

### Detection of epilepsy under ideal conditions

We first checked the proposed deep learning method by using the EEG signal without artifacts and noise. After EEG preprocessing (data segmentation and shaping), EEG would be fed into our deep neural network model, and its ultimate goal was to effectively learn EEG features and classify correctly.

#### Two-class detection task results

In the task of classification of epileptic EEG, the first category was to distinguish the normal EEG of healthy people and the epileptic EEG of epileptic patients. In our public dataset, it belonged to the classification between datasets (A, B) and datasets (C, D, E). However, some scholars believe that the non-epileptic interictal EEG should also be regarded as normal EEG in clinical practice. In our experiment, we mainly identified non-epileptic active EEG (datasets A, B and C, D) and epileptic seizure active EEG (dataset E).

Considering that each dataset has 100 signals, there is a class imbalance problem in our classification task. Based on these considerations, on the one hand, we considered data expansion, on the other hand, we selected non-epileptic EEG randomly in our experiment. In addition, in our evaluation indicators, we no longer simply relied on specificity, the sensitivity and accuracy were also our concerns. The data in the Table 3 showed that the epilepsy EEG recognition model based on 1-DCNN and indRNN can effectively identify the disease. Regarding the identification experiment results of the two-class task, both Acc and Spec achieved 100%, which was the best performance in the baseline algorithms.

**Table 3:** Two-class detection task results

Method	Spec	Sen	Acc
LSTM	94.62	89.67	93.33
1DCNN	96.17	94.37	95.36
INDRNN	93.57	91.57	93.52
RESNET(1DCNN)	98.69	96.78	97.47
RCNN	**100**	**97**.**50**	**100**

#### Three-class detection task results

Non-epileptic EEG can be divided into normal EEG and interictal EEG, so we have made a three-class classification task. The three-class classification task was mainly to distinguish different brain electrical activities: normal, interictal and ictal. In addition to the automatic diagnosis of patients, the three-class model can also detect the symptoms of epilepsy patients. Such a model was more meaningful. Table 4 showed the results under our evaluation indicators. As shown in the Table 4, our algorithm achieves the best results in terms of specificity, sensitivity and accuracy in the baseline algorithm.

**Table 4:** Three-class detection task results

Method	Spec	Sen	Acc
LSTM	89.58	90.42	91.26
1DCNN	94.87	89.43	93.82
INDRNN	92.68	90.67	91.53
RESNET(1DCNN)	98.28	96.50	97.79
RCNN	**100**	**98**.**48**	**100**

### Detection epilepsy under real conditions

There are noise and interference in the real world data, such as muscle activity, eye movement interference and environmental noise. The model’s performance in the real world can bring a real significance to clinical practice. Considering these, our experiment collected EEG data of 15 patients in a clinical environment, which were divided into interictal period, preictal period and ictal period according to the existing definition of epilepsy stage.

#### Two-class detection task results

For patients in the real world, it is more meaningful to predict the onset of seizures. The premise of accurately predicting seizures is the accurate recognition of EEG in the pre-seizure period. Different from the dichotomous experiment under the ideal condition, we no longer pay attention to non-epileptic activity EEG and seizure activity EEG. We are more concerned about the difference between preictal EEG and ictal EEG. We used 15 patients with pre-onset and onset EEG for the experiment and the results were shown in Table 5. Epilepsy was the manifestation of the disease, the real-world data have interference, the corresponding results have been reduced, but still in the baseline method is the best.

**Table 5:** Two-class detection task results

Method	Spec	Sen	Acc
LSTM	84.79	83.24	85.64
1DCNN	89.58	84,89	88.73
INDRNN	85.58	85.63	83.41
RESNET(1DCNN)	89.79	88.76	90.57
RCNN	**91**.**42**	**86**.**58**	**90**.**74**

#### Three-class detection task results

The definition of the epilepsy periods has been introduced above, which are interictal, preictal and ictal. On the basis of the two-class classification model, the task of interictal EEG recognition was added. Recognition of preictal can provide a reference for seizure prediction model, and recognition of the interictal period has a positive effect on reducing the false alarm rate of epileptic seizure prediction. Table 6 showed the results of three classification experiments. Our method is still the best among the baseline methods.

**Table 6:** Three-class detection task results

Method	Spec	Sen	Acc
LSTM	85.54	82.38	84.47
1DCNN	86.39	85.35	87.39
INDRNN	83.56	86.73	84.65
RESNET(1DCNN)	89.93	87.48	91.83
RCNN	**90**.**61**	**85**.**42**	**92**.**11**

### Comparison of research results of the same category

In addition to the comparison with the baseline method mentioned above, we also compared the results of similar studies in recent years, as shown in Table 7. For the private data used in this experiment, the dataset in the same category study is already a variable, so it is not considered in this comparison. We have collected result from those studies based on the University of Bourne data set over the past five years, including two classifications and three classifications. From the evaluation index of the model, our research had achieved outstanding specificity, sensitivity and accuracy. Only in the three classification problems, the sensitivity of our algorithm was lower than that of Behara et al. [36]

**Table 7:** Comparison with similar studies results

Method	Classifier	Dataset	Task	Spec	Sen	Acc
Jaiswal and Banka (2017) [37]	ANN	University of Bonn	Two categories	98.30	98.82	98.72
Wang et al. (2017) [38]	SVM	University of Bonn	Two categories	97.98	99.56	99.25
Acharya et al. (2012) [39]	GMM	University of Bonn	Three categories	99.00	99.00	99.00
Behara et al. (2016) [36]	LSSVM	University of Bonn	Three categories	96.96	**99**.**66**	97.19
Proposed	RCNN	University of Bonn	Three categories	**100**	97.50	**100**
Proposed	RCNN	University of Bonn	Three categories	**100**	98.48	**100**

## Conclusions

The professional neurologists’ reading of EEG is the main method to determine epilepsy. However, it is complex and time-consuming to observe and detect long-range EEG signals by people. Most of the current researches on EEG diagnosis of epilepsy use single-lead data, and the researches on multi-lead and full-lead data need to be further improved. In this paper, we propose the RCNN model to achieve the automatic diagnosis task of epilepsy EEG, and achieve the automatic labeling of different stages of epilepsy EEG.Our research describes a new method of automatic detection of epilepsy that can directly process the original EEG. In the framework of the traditional residual network, we used the one-dimensional convolutional neural network to replace it, and combined independent convolution recurrent neural network to form a new recurrent residual network to realize the automatic diagnosis of epileptic EEG. Firstly, we used the residual network architecture of the one-dimensional convolutional neural network to learn important features of EEG, and then the recurrent neural network was used to learn the relationship between sequences. Finally, the classification results were output. Our research demonstrated the potential of deep learning in epilepsy detection and seizure prediction, and the possibility of combining convolutional neural networks with recurrent neural networks. It is hoped that this study can promote the further development of epileptic seizure prediction system.

## Data Availability

All data were collected from the patients who had been admitted to the hospital, Because of the hospital’s regulations, the data cannot be made publicly available.

## Abbreviations

EEG: Electroencephalogram
1-D: One-dimensional
2-D: Two-dimensional
3-D: Three-dimensional
indRNN: Independent recurrent neural network
CNN: Convolution neural network
RCNN: Residual network architecture-independent convolutional recurrent neural network
RNN: Recurrent neural network
GRU: Gate recurrent unit
LSTM: Long short term memory
bi-LSTM: Bi-directional long short-term memory
LRCN: Long-term recursive convolutional network
E3D-LSTM: Eidetic 3D long short-term memory
SGD: Stochastic gradient descent
Sens: Sensitivity
Spe: Specificity
Acc: Accuracy
TP: True positive
FP: False positive
TN: True negative
FN: False negative
F: Female
M: Male
SPS: Simple partial
CPS: Complex partial
IT: Ictal time
BN: Batch normalization
ANN: Artificial neural network
SVM: Support vector machines
GMM: Gaussian mixture model
LSSVM: LS support vector machines

## References

[1] Gonzalez Otarula KA, Mikhaeil-Demo Y, Bachman EM, Balaguera P, Schuele S (2019). Automated seizure detection accuracy for ambulatory EEG recordings. Neurology.

[2] Acharya UR, Molinari F, Sree SV, Chattopadhyay S, Ng K-H, Suri JS (2012). Automated diagnosis of epileptic EEG using entropies. Biomed Signal Process Control.

[3] Cook MJ, O’Brien TJ, Berkovic SF, Murphy M, Morokoff A, Fabinyi G, D’Souza W, Yerra R, Archer J, Litewka L, Hosking S, Lightfoot P, Ruedebusch V, Sheffield WD, Snyder D, Leyde K, Himes D (2013). Prediction of seizure likelihood with a long-term, implanted seizure advisory system in patients with drug-resistant epilepsy: a first-in-man study. Lancet Neurol.

[4] Hossain MS, Amin SU, Alsulaiman M, Muhammad G (2019). Applying deep learning for epilepsy seizure detection and brain mapping visualization. ACM Trans Multimed Comput Commun Appl.

[5] Omidvarnia A, Kowalczyk MA, Pedersen M, Jackson GD (2019). Towards fast and reliable simultaneous EEG-FMRI analysis of epilepsy with automatic spike detection. Clin Neurophysiol.

[6] Seneviratne U, Karoly P, Freestone DR, Cook MJ, Boston RC (2019). Methods for the detection of seizure bursts in epilepsy. Front Neurol.

[7] Namazi H, Kulish VV, Hussaini J, Hussaini J, Delaviz A, Delaviz F, Habibi S, Ramezanpoor S (2016). A signal processing based analysis and prediction of seizure onset in patients with epilepsy. Oncotarget.

[8] Hannun AY, Rajpurkar P, Haghpanahi M, Tison GH, Bourn C, Turakhia MP, Ng AY (2019). Cardiologist-level arrhythmia detection and classification in ambulatory electrocardiograms using a deep neural network. Nat Med.

[9] Tsiouris KM, Pezoulas VC, Zervakis M, Konitsiotis S, Koutsouris DD, Fotiadis DI (2018). A long short-term memory deep learning network for the prediction of epileptic seizures using EEG signals. Comput Biol Med.

[10] Shi XJ, Chen ZR, Wang H, Yeung DY, Wong WK, Woo WC. Convolutional LSTM network: a machine learning approach for precipitation nowcasting. Adv Neural Inf Process Syst. 2015;28.

[11] Bou Assi E, Nguyen DK, Rihana S, Sawan M (2017). Towards accurate prediction of epileptic seizures: a review. Biomed Signal Process Control.

[12] Zhang Y, Guo Y, Yang P, Chen W, Lo B (2019). Epilepsy seizure prediction on EEG using common spatial pattern and convolutional neural network. IEEE J Biomed Health Informat.

[13] Bayoumi M. Epileptic seizure detection using deep convolutional autoencoder. In: IEEE workshop on signal processing systems.

[14] Ozcan AR, Erturk S (2019). Seizure prediction in scalp EEG using 3D convolutional neural networks with an image-based approach. IEEE Trans Neural Syst Rehabil Eng.

[15] Truong ND, Nguyen AD, Kuhlmann L, Bonyadi MR, Yang JW, Ippolito S, Kavehei O (2018). Convolutional neural networks for seizure prediction using intracranial and scalp electroencephalogram. Neural Netw.

[16] Van Leeuwen K, Sun H, Tabaeizadeh M, Struck A, Van Putten M, Westover M (2019). Detecting abnormal electroencephalograms using deep convolutional networks. Clin Neurophysiol.

[17] Wei XY, Zhou L, Chen ZY, Zhang LJ, Zhou Y (2018). Automatic seizure detection using three-dimensional CNN based on multi-channel EEG. BMC Med Informat Decision Making.

[18] Golmohammadi M, Ziyabari S, Shah V, Von Weltin E, Campbell C, Obeid I, Picone J. Gated recurrent networks for seizure detection. In: IEEE signal processing in medicine and biology symposium.

[19] Golmohammadi M, Ziyabari S, Shah V, Weltin EV, Campbell C, Obeid I, Picone J. Gated recurrent networks for seizure detection. In: Signal processing in medicine and biology symposium (SPMB) 2018.

[20] Ma X, Qiu S, Zhang Y, Lian X, He H. Predicting epileptic seizures from intracranial EEG using LSTM-based multi-task learning. In: Chinese conference on pattern recognition and computer vision (PRCV). Springer; 2018. p. 157–67.

[21] Talathi SS. Deep recurrent neural networks for seizure detection and early seizure detection systems. 2017. arXiv preprint arXiv:1706.03283.

[22] Li S, Li WQ, Cook C, Zhu C, Gao YB. Independently recurrent neural network (INDRNN): building a longer and deeper RNN. In: 2018 IEEE/CVF Conference on Computer Vision and Pattern Recognition (CVPR). 2018;5457–66. 10.1109/Cvpr.2018.00572.

[23] Lipton ZC, Kale DC, Elkan C, Wetzel R. Learning to diagnose with LSTM recurrent neural networks. 2015. arXiv preprint arXiv:1511.03677.

[24] Subasi A, Kevric J, Canbaz MA (2019). Epileptic seizure detection using hybrid machine learning methods. Neural Comput Appl.

[25] Ahmedt-Aristizabal D, Fookes C, Denman S, Nguyen K, Sridharan S, Dionisio S (2019). Aberrant epileptic seizure identification: a computer vision perspective. Seizure-Eur J Epilepsy.

[26] Golmohammadi M, Ziyabari S, Shah V, de Diego SL, Obeid I, Picone JJapa. Deep architectures for automated seizure detection in scalp EEGS. 2017.

[27] Wei XY, Zhou L, Zhang Z, Chen ZY, Zhou Y (2019). Early prediction of epileptic seizures using a long-term recurrent convolutional network. J Neurosci Methods.

[28] Wang Y, Jiang L, Yang M, Li L, Long M, Feifei L. Eidetic 3D LSTM: A model for video prediction and beyond. In: International conference on learning representations

[29] Andrzejak RG, Lehnertz K, Mormann F, Rieke C, David P, Elger CEJPRE (2002). Indications of nonlinear deterministic and finite dimensional structures in time series of brain electrical activity: Dependence on recording region and brain state. Phys Rev E Stat Nonlinear Soft Matter Phys.

[30] Acharya UR, Hagiwara Y, Adeli H (2018). Automated seizure prediction. Epilepsy Behav.

[31] Li FL, Liang Y, Zhang LY, Yi CL, Liao YY, Jiang YL, Si YJ, Zhang YS, Yao DZ, Yu L, Xu P (2019). Transition of brain networks from an interictal to a preictal state preceding a seizure revealed by scalp eeg network analysis. Cogn Neurodyn.

[32] Nandy A, Alahe MA, Uddin SN, Alam S, Nahid A-A, Awal MA. Feature extraction and classification of EEG signals for seizure detection. In: 2019 International Conference on Robotics, Electrical and Signal Processing Techniques (ICREST). IEEE. p. 480–485.

[33] Trinka E, Kalviainen R (2017). 25 years of advances in the definition, classification and treatment of status epilepticus. Seizure.

[34] Zhang M, Li W, Du Q (2018). Diverse region-based CNN for hyperspectral image classification. IEEE Trans Image Process.

[35] Zhang ZX, Liu QJ, Wang YH (2018). Road extraction by deep residual u-net. IEEE Geosci Remote Sens Lett.

[36] Behara DST, Kumar A, Swami P, Panigrahi BK, Gandhi T. Detection of epileptic seizure patterns in EEG through fragmented feature extraction. In: International Conference on Computing for Sustainable Global Development, p. 2539–2542.

[37] Jaiswal AK, Banka H (2017). Local pattern transformation based feature extraction techniques for classification of epileptic EEG signals. Biomed Signal Process Control.

[38] Wang L, Sun S, Zhang B, Yang L, Yao Y, Zhuang X, Chen Y (2017). Viologen-based conjugated ionic polymer for nonvolatile rewritable memory device. Eur Polymer J.

[39] Acharya UR, Sree SV, Alvin APC, Suri JS (2012). Use of principal component analysis for automatic classification of epileptic EEG activities in wavelet framework. Expert Syst Appl.

